# Prevalence and Risk of Carpal Tunnel Syndrome in Parkinson’s Disease: A Systematic Review and Meta-Analysis

**DOI:** 10.3390/jfmk11010066

**Published:** 2026-02-02

**Authors:** Amir N. Attia, Kareem Wael Raafat, Mohamed R. Ezz, Ehab Naser Sabry, Mariam M. Mohammed, Ahmed M. Amin, Mohamed S. Syed, George M. Pamboris, Spyridon Plakias, Frederic Viseux, Ismail A. Ibrahim

**Affiliations:** 1 Kasr Alainy School of Medicine, Cairo University, Cairo 11562, Egypt; amirnabiell@gmail.com (A.N.A.); ahmeed.am2003@gmail.com (A.M.A.); 2 Global Alliance of Young Researchers, Istanbul 34000, Turkey; kareemwaelraafat@gmail.com (K.W.R.); mohamedredaezz.offical@gmail.com (M.R.E.); ehab.naser69@gmail.com (E.N.S.); mariammahmoudmohammed06@gmail.com (M.M.M.); mos333818@gmail.com (M.S.S.); ismailaiaibrahim@gmail.com (I.A.I.); 3 Faculty of Medicine, Suez Canal University, Ismailia 41522, Egypt; 4 Faculty of Medicine, Tanta University, Tanta 31527, Egypt; 5 Faculty of Physical Therapy, Misr University of Science and Technology, Giza 12566, Egypt; 6 Faculty of Pharmacy, Fayoum University, Fayoum 63514, Egypt; 7 Faculty of Medicine, Ain Shams University, Cairo 11566, Egypt; 8 Department of Health Sciences, European University Cyprus, Nicosia 2404, Cyprus; 9 Department of Physical Education and Sport Science, University of Thessaly, 42132 Trikala, Greece; spyros_plakias@yahoo.gr; 10 Department of Medicine, General Hospital of Hazebrouck, F-59190 Hazebrouck, France; frederic.viseux@uphf.fr; 11 Université Polytechnique Hauts-de-France, LAMIH, CNRS, UMR 8201, F-59313 Valenciennes, France; 12 Faculty of Health Sciences, Fenerbahçe University, Istanbul 34758, Turkey

**Keywords:** Parkinson’s disease, carpal tunnel syndrome, entrapment neuropathy, prevalence

## Abstract

**Background**: Parkinson’s disease (PD) is a progressive neurodegenerative disorder characterised by motor and non-motor symptoms. Several studies have reported varying prevalence of Carpal Tunnel Syndrome (CTS) among individuals with PD. **Objective**: This study aimed to estimate the pooled prevalence of CTS in people with PD and explore any potential association between the two conditions. **Methods**: This systematic review and meta-analysis was conducted and reported in accordance with the PRISMA 2020 guidelines. A systematic search was performed across PubMed, the Cochrane Central Register of Controlled Trials (CENTRAL), Web of Science (WoS), Scopus, and EMBASE from inception to April 2024. Studies reporting CTS prevalence data in individuals with PD were included. Methodological quality was assessed using the National Institutes of Health (NIH) quality assessment tool. Pooled prevalence estimates were calculated using a random-effects model. Risk difference (RD) and risk ratio (RR) were calculated to assess the association between PD and CTS compared with control groups. **Results**: A total of 7 studies involving 411 participants (343 with PD and 68 controls) met the inclusion criteria, with 679 wrists assessed. The pooled prevalence of CTS in PD was estimated at 15% (95% CI: 0.07–0.28) with significant heterogeneity (*p* < 0.001, I^2^ = 91%). The RD was 10% (95% CI: 0.04–0.16, *p* = 0.002), with low heterogeneity (*p* = 0.29, I^2^ = 19%). The RR of CTS in PD compared with controls was 3.31 (95% CI: 0.60–18.42, *p* = 0.17), with moderate heterogeneity (*p* = 0.13, I^2^ = 52%). **Conclusions**: This meta-analysis provides preliminary pooled estimates indicating a potentially increased prevalence of carpal tunnel syndrome in individuals with PD. Although the findings suggest a possible association, clinicians should maintain increased vigilance for CTS symptoms in patients with PD presenting with upper-limb sensory or motor complaints. From a biomechanical and functional perspective, these findings highlight the importance of routine upper-limb screening and the implementation of rehabilitation strategies targeting hand use, dexterity, and sensorimotor control within physiotherapy practice. Further high-quality studies with larger, well-characterised samples are required to confirm this relationship and clarify its clinical and functional implications.

## 1. Introduction

Carpal tunnel syndrome (CTS), the most common entrapment neuropathy of the upper limb, results from compression of the median nerve and typically presents with pain, paresthesia, numbness, and weakness in its anatomical distribution [1,2,3]. Epidemiological studies estimate the prevalence of CTS in the general population to be approximately 3–6% [4,5], although some reports suggest that the lifetime risk may be as high as 10% [6]. Accounting for nearly 90% of all upper limb entrapment neuropathies, CTS contributes substantially to upper extremity disability [7]. Beyond physical symptoms, CTS can impair hand function, reduce quality of life, and increase healthcare utilisation, underscoring the importance of early diagnosis and timely intervention [8]. First-line management typically involves conservative management such as wrist splinting, activity modification, and corticosteroid injections; for refractory or severe cases, surgical decompression via release of the transverse carpal ligament is required and offers sustainable symptom relief [9,10,11].

Given their overlapping symptomatology, recent literature has explored whether Parkinson’s disease (PD) may predispose individuals to CTS. Recent studies have suggested a potential association between PD and an increased risk of developing CTS, particularly among individuals with tremor-dominant PD. This PD subtype may predispose patients to median nerve compression due to postural abnormalities and repetitive tremor-related movements [12]. PD affects approximately 1–2% of individuals over the age of 60, making it a common neurodegenerative disorder [13]. It is marked by the loss of dopaminergic neurons in the substantia nigra [14] and results in key motor symptoms, such as tremor, rigidity, and bradykinesia [14,15], as well as postural instability [16] and altered pain processing [17]. In addition to tremor-related mechanical stress, PD is also characterised by increased muscle tone, altered sensorimotor integration, and compensatory upper-limb postures, which may collectively increase loading on the median nerve [16,17,18]. Although the precise pathophysiological mechanisms linking PD and CTS remain unclear, several hypotheses have been proposed, including alterations in nerve conduction velocity, changes in musculoskeletal biomechanics, and systemic inflammation, all of which could predispose to median nerve compression in the carpal tunnel [12]. Moreover, abnormal forearm co-contraction, reduced neural mobility, and altered proprioceptive control in PD may further contribute to carpal tunnel loading and nerve irritation [17,19,20].

While CTS is primarily diagnosed clinically, it is often misdiagnosed due to misconceptions surrounding its characteristic signs and symptoms [21]. The potential association between PD and CTS remains under investigation, with emerging evidence suggesting shared or overlapping pathophysiological mechanisms. A recent systematic review and meta-analysis by Atwan et al. [7] reported that individuals with PD often exhibit increased median nerve cross-sectional area on ultrasonographic evaluation and a higher prevalence of CTS than control groups, despite minimal differences in electrophysiological findings. Nevertheless, the overall relationship between CTS and PD remains inconclusive.

However, the review by Atwan et al. [7] focused exclusively on ultrasonographic nerve morphology and did not examine the epidemiological relationship between PD and clinically diagnosed CTS. Their analysis included only three small studies and did not address CTS prevalence, relative risk, diagnostic confirmation, or the functional implications of CTS within PD populations. In contrast, the present review extends beyond structural nerve changes to synthesise pooled prevalence and risk estimates using both electrodiagnostic and ultrasonographic data, evaluate potential sources of heterogeneity, and integrate neuromechanical and physiotherapy-relevant mechanisms. This broader perspective provides a more clinically meaningful understanding of CTS in PD and offers practical insights directly applicable to rehabilitation practice.

From a biomechanical perspective, CTS in Parkinson’s disease may further compromise manual dexterity, grip force modulation, and sensorimotor integration, all of which are already impaired due to central motor dysfunction [16,17,19]. These functional consequences position CTS not merely as a comorbidity, but as a potential modifier of upper-limb movement performance and task execution in individuals with PD.

Nevertheless, the overall relationship between CTS and PD remains inconclusive. Therefore, this systematic review and meta-analysis aims to synthesise current evidence on the prevalence of CTS among individuals with PD and to evaluate the relative risk of CTS compared with controls. By addressing inconsistencies in the literature, this review seeks to inform future screening and management strategies. A better understanding of this relationship may facilitate earlier recognition and targeted management of CTS in PD, including neuromuscular assessment, ergonomic education, and sensorimotor rehabilitation. Clarifying this relationship may help physiotherapists and rehabilitation clinicians more effectively identify and manage upper limb dysfunction in people living with PD.

## 2. Materials and Methods

### 2.1. Protocol and Guidelines

This systematic review and meta-analysis was conducted following the guidelines outlined in the Cochrane Handbook [22] and reported according to the Preferred Reporting Items for Systematic Reviews and Meta-Analyses (PRISMA) 2020 statement [23]. The review protocol was preregistered in the International Prospective Register of Systematic Reviews (PROSPERO) (Registration number: CRD42024535945). The full protocol can be accessed at: https://www.crd.york.ac.uk/prospero/display_record.php?ID=CRD42024535945. No amendments to the registered protocol were made. The eligibility criteria were established using the Population, Intervention, Comparison, Outcome, and Study (PICOS) framework [24] and were defined as follows:

- **Population (P):** Studies involving individuals of any age diagnosed with PD were eligible.
- **Intervention (I):** Studies that assessed and diagnosed CTS in patients with PD.
- **Comparison (C):** Studies that compared control groups with or without PD patients (when available).
- **Outcome (O):** Studies were considered eligible if they included the prevalence of CTS in PD patients. Studies were eligible if CTS was diagnosed clinically or via electrodiagnostic or ultrasound-based criteria, reflecting real-world diagnostic variability and allowing inclusion of clinically relevant data.
- **Study Design (S):** Observational studies, including cross-sectional, case–control, and cohort designs, were included.

Exclusion criteria included clinical trials, case reports, case series, letters, editorials, review articles, non-English publications, studies lacking sufficient data on CTS prevalence, and studies not involving human subjects. Conference abstracts were included if they contained sufficient methodological detail to allow data extraction and risk-of-bias assessment.

### 2.2. Search Methods

A comprehensive electronic search was conducted across the following databases: PubMed, Scopus, EMBASE, Web of Science (WoS) and Cochrane Central Register of Controlled Trials (CENTRAL) from the inception of each database to April 2024. To ensure thoroughness, both forward and backwards citation tracking of the included studies was performed to identify any relevant articles that were not captured in the initial search. The full electronic search strategies for all databases, including search terms, Boolean operators, and applied limits, are provided in Supplementary File S1.

### 2.3. Study Selection

Articles retrieved from the database search were imported into Mendeley reference manager version 2.116.0 (Elsevier, Amsterdam, The Netherlands) and duplicate records were removed. Two reviewers independently screened titles, abstracts, and full texts. Discrepancies were resolved through discussion or with the assistance of a third reviewer. No automation tools were used in the screening process.

### 2.4. Data Extraction

Data was independently extracted by two authors using a standardised Microsoft Excel 365 spreadsheet (Microsoft Corporation, Redmond, WA, USA). Extracted information included the first author’s surname, year of publication, study location, study design, sample size, population characteristics (e.g., mean age, gender distribution, Unified Parkinson’s Disease Rating Scale (UPDRS) part III score, tremor severity, Hoehn and Yahr stage), prevalence of CTS, outcome measures at baseline, post-observation, and follow-up, as well as study limitations. A third reviewer resolved any discrepancies. No missing data were encountered; therefore, contacting the study authors was not required.

### 2.5. Quality Assessment and Risk of Bias

The methodological quality of the included studies was independently assessed by two reviewers using the NIH quality assessment tool for observational cohort and cross-sectional studies [25]. This tool evaluated 14 criteria, including the clarity of the research question, definition and selection of the study population, recruitment method, justification of sample size, temporal relationship between exposure and outcome, adequacy of follow-up, and reliability of outcome measurement. The NIH tool used for quality assessment considered studies scoring ≥ 8 as “good” quality, 6–7 as “fair,” and <6 as “poor”. Any discrepancies were resolved through discussion, and a senior author was consulted when consensus could not be reached.

### 2.6. Data Synthesis and Analysis

Prevalence data were extracted as event counts and corresponding totals. Assessment of reporting bias (e.g., funnel plot or Egger’s test) was not feasible because fewer than 10 studies were available. Meta-analyses were performed using R software (version 4.3.3; R Foundation for Statistical Computing, Vienna, Austria) with the ‘meta’ and ‘metafor’ packages [26,27,28]. A random-effects meta-analysis was used to estimate the pooled prevalence of CTS in patients with PD, along with 95% confidence intervals (CIs). Statistical heterogeneity was evaluated using the I^2^ and the chi-square test. A *p*-value of <0.05 was considered statistically significant, while an I^2^ > 50% indicated substantial heterogeneity. In such cases, a sensitivity analysis was conducted by removing one study at a time to assess the impact on the overall effect estimate. Risk Difference (RD) and Risk Ratio (RR) were calculated using Review Manager software (RevMan version 5.4.1; The Nordic Cochrane Centre, Copenhagen, Denmark). A formal certainty assessment (e.g., GRADE) was not conducted due to the limited number of studies and the observed heterogeneity.

## 3. Results

### 3.1. Literature Search and Study Selection

**Figure 1:**
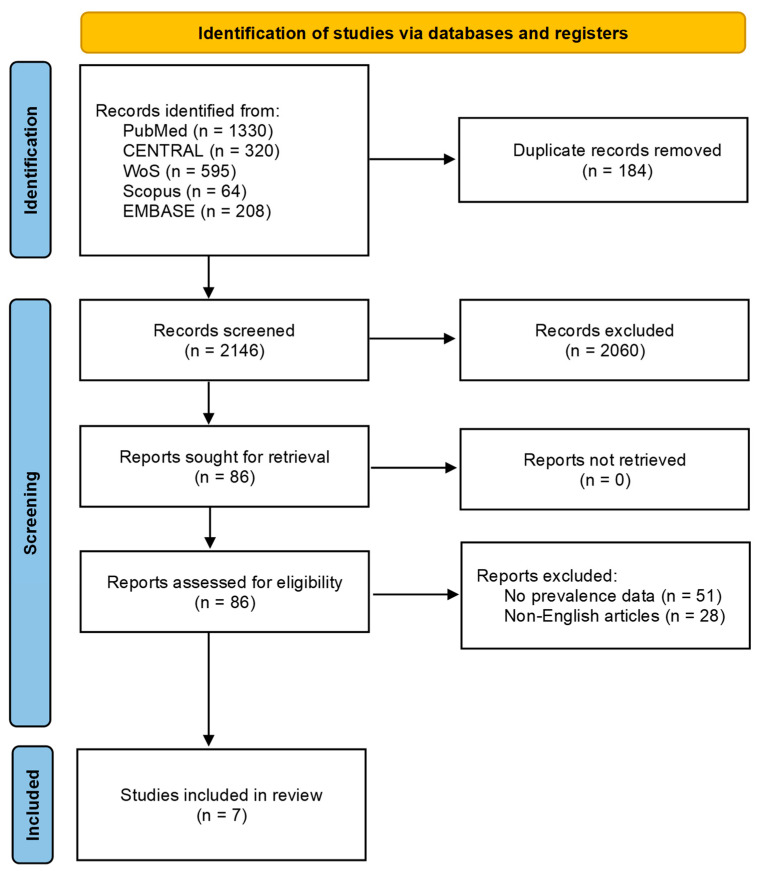
PRISMA flow diagram with search protocol summary.

The initial database search yielded 2330 potentially relevant records. After removal of 184 duplicates, 2146 records remained and were screened by title and abstract. Of these, 86 articles were deemed potentially eligible and underwent full-text review. Of these, 79 articles were excluded due to the absence of relevant CTS prevalence data in individuals with PD or inclusion of non-relevant populations (*n* = 51), or because they were published in a non-English language (*n* = 28). A total of seven studies met the eligibility criteria and were included in this systematic review and meta-analysis [12,29,30,31,32,33,34]. A PRISMA 2020 flow diagram outlining the selection process is presented in Figure 1.

### 3.2. Study Characteristics and Participants

Characteristics of the included studies are depicted in Table 1. The seven eligible studies, published between 2007 and 2021, included a total of 411 participants, of whom 343 had been diagnosed with PD, and 68 were included as controls in three of the included studies [31,32,33]. The mean age of PD participants ranged from 64 ± 3.72 (mean ± standard deviation) years [29] to 71.2 ± 8.6 years [30], while in the control groups, the mean age ranged from 60.25 ± 14.67 [31] to 62.03 ± 10.4 years [32]. The proportion of male participants in the PD groups varied from 34.1% to 60.6%. There was marked variability in reported CTS prevalence rates across studies and countries. Three studies were conducted in Turkey and reported prevalence rates ranging from 16.13% to 38.73% [30,32,33]. In the USA, the prevalence of CTS in PD was reported to be 15.91% [34], whereas a French study reported a lower prevalence of 5.22% [29]. Two studies conducted in South Korea reported prevalence rates between 7.32% and 10.61% [12,31]. Most studies used either the American Association of Electrodiagnostic Medicine criteria or a combination of clinical signs and electromyography to diagnose CTS.

### 3.3. Outcome Measures

Three studies used the Hoehn and Yahr (H&Y) scale to assess PD symptom severity. The mean H&Y stage scores were reported as follows: 1.4 ± 0.62 [12], 2.73 ± 0.94 [32], and 2.27 ± 0.80 [31].

### 3.4. Methodological Quality

The National Institutes of Health (NIH) quality assessment tool was used to evaluate the methodological quality of the included studies. One study [31] received a score of 6, categorised as fair, indicating moderate quality. Two studies [12,32] scored 7 and were also classified as fair, reflecting moderate quality. The remaining four studies [29,30,33,34] received a score of 8, placing it in the good category and indicating a low risk of bias. Overall, four of the seven studies were rated as having good quality and a low risk of bias, while the remaining three were rated as fair quality, indicating a moderate risk of bias.

### 3.5. Pooled Prevalence of CTS in PD

**Figure 2:**
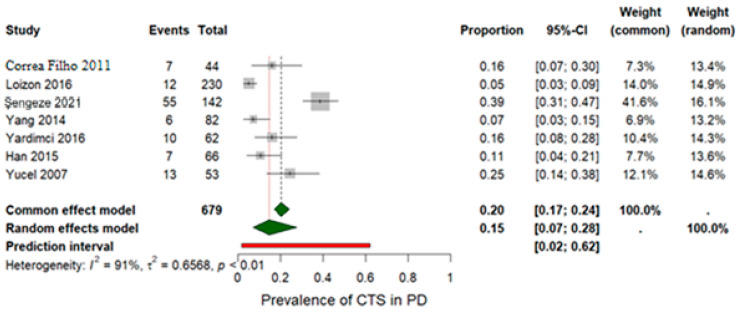
Forest plot showing the pooled prevalence of CTS in individuals with PD based on wrist-level data from seven studies [12,29,30,31,32,33,34].

**Figure 3:**
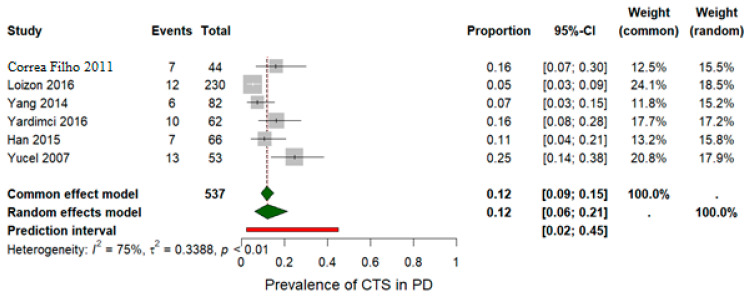
Sensitivity analysis excluding the study by Şengeze et al. [30], which contributed to heterogeneity. Exclusion of this study reduced the pooled prevalence estimate to 12% and lowered heterogeneity (*I^2^* = 75%). The analysis is based on the studies included in the pooled prevalence estimate [12,29,31,32,33,34].

The pooled prevalence of CTS among examined wrists was estimated at 20% based on wrist-level data from seven studies that included a total of 679 wrists assessed via electromyography, as shown in Table 2. Because several studies reported CTS per wrist rather than per patient, and others combined both approaches, wrist-level prevalence was used for consistency and comparability across datasets. Using a random-effects model, the pooled prevalence was slightly lower at 0.15 (95% CI: [0.07; 0.28]) (Figure 2). Substantial statistical heterogeneity was detected, as indicated by an *I^2^* value of 91% and a *p*-value < 0.001. A sensitivity analysis was performed by excluding the study by Şengeze et al. [30], which was suspected to contribute to the heterogeneity (Figure 3). This exclusion did not substantially reduce heterogeneity (*p* = 0.001; *I^2^* = 75%), but it did affect the pooled prevalence, which was reduced to 12% under both the random-effects model (REM) and the fixed-effects model (FEM). These findings underscore the clinical relevance of CTS as a potential comorbidity in individuals with PD.

### 3.6. Risk Difference Analysis

**Figure 4:**
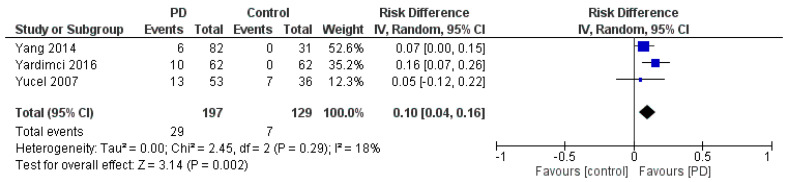
Forest plot of the RD for CTS in patients with PD compared with controls, based on three studies [31,32,33].

Three studies were included to estimate the RD analysis (Figure 4) [31,32,33]. The pooled RD for CTS in PD was calculated to be 0.10 with 95% CI [0.04, 0.16], indicating a statistically significant difference in CTS prevalence between PD and control groups. The pooled studies were homogeneous (*p* = 0.29, *I^2^* = 19%). Additionally, the test for the overall effect yielded a Z-value of 3.14 (*p* = 0.002), suggesting a significant association between PD and CTS.

### 3.7. Risk Ratio Analysis

**Figure 5:**
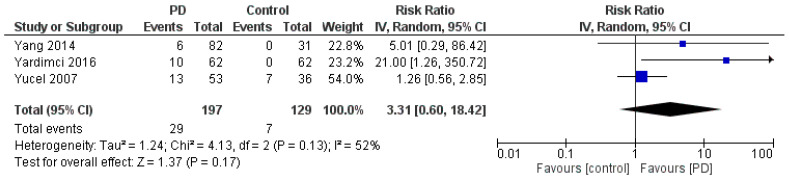
Forest plot of RR for CTS in patients with PD compared with control groups, based on three studies [31,32,33]. Arrows indicate confidence intervals extending beyond the plotted axis range.

**Figure 6:**
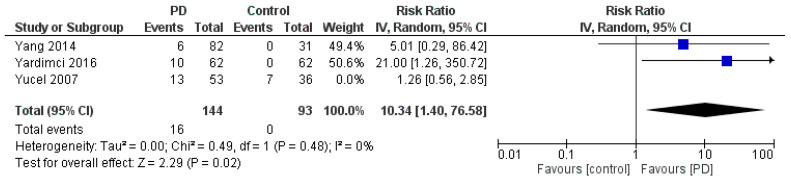
Sensitivity analysis of the RR for CTS in patients with PD compared with controls, based on studies including control groups [31,32,33].

To estimate the RR, data from the three studies that included both PD and control groups were used [31,32,33] (Figure 5). The pooled RR for CTS in individuals with PD was 3.31 (95% CI: [0.60, 18.42]), suggesting that patients with PD may be more than three times as likely to develop CTS compared to controls. However, this result was not statistically significant (*p* = 0.17), and the analysis showed moderate heterogeneity (*I^2^* = 52%, *p* = 0.13). The wide confidence interval further suggests the uncertainty in the effect estimate; therefore, this potential association should be interpreted with caution. Assessment of publication bias was not performed because fewer than 10 studies were available for each outcome. A sensitivity analysis of the RR was also conducted to assess the robustness of the pooled estimate (Figure 6). Excluding individual studies did not materially alter the direction of the effect, although the confidence intervals remained wide, reflecting the limited number of available studies and underlying heterogeneity.

### 3.8. Meta-Regression, Reporting Bias, and Certainty of Evidence

Meta-regression analyses to explore potential sources of heterogeneity were not feasible due to the small number of included studies (*n* = 7), only three of which included control groups. Similarly, assessment of reporting bias using funnel plots or Egger’s test was not performed because fewer than ten studies were available, in accordance with methodological recommendations. Consequently, the ability to formally explore sources of heterogeneity and publication bias was limited. A formal assessment of the certainty of evidence (e.g., using GRADE) was not conducted due to the limited number of studies and substantial heterogeneity; therefore, the findings should be interpreted with caution.

## 4. Discussion

This study aimed to systematically review and synthesise available evidence on the prevalence of CTS in individuals with PD and to determine whether PD is linked to an increased risk of CTS compared to a control population without PD. By conducting a meta-analysis using pooled prevalence data and estimating both RD and RR, we aimed to clarify the potential relationship between these two conditions. Our pooled analysis revealed an overall prevalence of 15% for CTS in individuals with PD, although this estimate should be interpreted with caution given the substantial heterogeneity observed among studies (*I^2^* = 91%). A significant RD of 10% (95% CI [0.04, 0.16], *p* = 0.002) was found when comparing PD patients to controls, suggesting a higher absolute prevalence of CTS in this population. Although the estimated relative risk (RR = 3.31, 95% CI [0.60, 18.42], *p* = 0.13) did not reach statistical significance, it indicated a trend toward increased risk. Substantial heterogeneity was observed in the prevalence estimates (*I^2^* = 91%), and moderate heterogeneity was noted in the RR analysis (*I^2^* = 52%). Beyond epidemiological estimates, these findings are clinically relevant because CTS may exacerbate impairments in hand function, manual dexterity, and fine motor control, thereby compounding pre-existing upper-limb movement limitations associated with Parkinson’s disease [8,16,17].

The reported prevalence of CTS among individuals with PD varied substantially across included studies, ranging from 5.2% to 38.7%. This variability may reflect differences in diagnostic criteria, assessment methods (e.g., ultrasonography vs. electrodiagnostic testing), study settings, or participant demographics. For example, studies employing sonographic assessments tended to report higher prevalence estimates [33], while those using stricter electrodiagnostic criteria, such as those outlined by the American Association of Electrodiagnostic Medicine, reported lower rates [29].

Several mechanisms may explain the increased prevalence of CTS observed in individuals with PD in this meta-analysis. Tremor-related repetitive stress may contribute to microtrauma of the median nerve, potentially increasing its CSA and susceptibility to compression, as supported by recent sonographic meta-analyses [7]. However, the contribution of tremor to CTS in PD remains uncertain, as PD–specific studies report conflicting associations between tremor laterality and CTS occurrence, with several cases involving the non-tremulous hand rather than the tremulous limb [12,31]. In PD, rigidity and bradykinesia are consistently associated with abnormal co-contraction of agonist and antagonist muscles in the upper limb, as demonstrated by electromyographic and biomechanical studies [35]. When this co-contraction involves the pronator and supinator muscles of the forearm, it tends to maintain the limb in rotational positions, particularly full supination, which are known to increase intracarpal tunnel pressure [36]. Additionally, frequent co-activation of finger flexor muscles in PD further enlarges the tunnel contents, raising intracarpal pressure [37]. Pressures exceeding 30–50 mmHg have been shown to impair intraneural venous flow and promote compressive oedema [38]. Furthermore, recent MRI evidence indicates that forearm rotation shifts the median nerve within the carpal tunnel during pronation and supination [39]. Consequently, the persistent and poorly controlled rotational movements typical of PD likely subject the median nerve to repetitive shear forces against the flexor tendons and the transverse carpal ligament. Collectively, these findings support the notion that muscle tone dysregulation in PD may chronically increase compressive and shear stresses within the carpal tunnel, thereby increasing the risk of median nerve entrapment. This aligns with the higher incidence of CTS reported among individuals with PD [33].

Additional factors may further contribute to the susceptibility of individuals with PD to CTS. Extensive clinical and anatomical evidence indicates that shortening or hyperactivity of muscles such as the pectoralis minor and the scalene group can diminish the infraclavicular or costoclavicular spaces, thereby compressing components of the brachial plexus and inducing paraesthesia or pain in the upper limb [40,41]. The “double crush” hypothesis further suggests that proximal low-grade compression heightens vulnerability to distal entrapment [42,43]. In PD specifically, axial rigidity, reduced arm swing, and persistent co-contraction of scapulothoracic and cervical muscles may result in a shortened pectoralis minor muscle or an elevated first rib, facilitating serial compression along the median nerve pathway. This perspective aligns with the broader postural and sensorimotor framework in PD described by Viseux et al. [17], who highlight postural instability, altered sensory reweighting, and abnormal muscle recruitment, consistent with earlier descriptions of axial and segmental postural deformities in PD [44]. Similar mechanisms have been described in non-PD populations, in which forward head posture and shoulder protraction mechanically strain the median nerve, reducing its excursion [45], and in which both compression and shear forces contribute to CTS pathophysiology [45]. Furthermore, early-stage PD is also associated with peripheral neuropathy affecting both small- and large-fibre nerves, possibly linked to hyperhomocysteinemia and altered vitamin B12 metabolism, which may impair nerve function and regeneration [46,47], further contributing to susceptibility.

**Figure 7:**
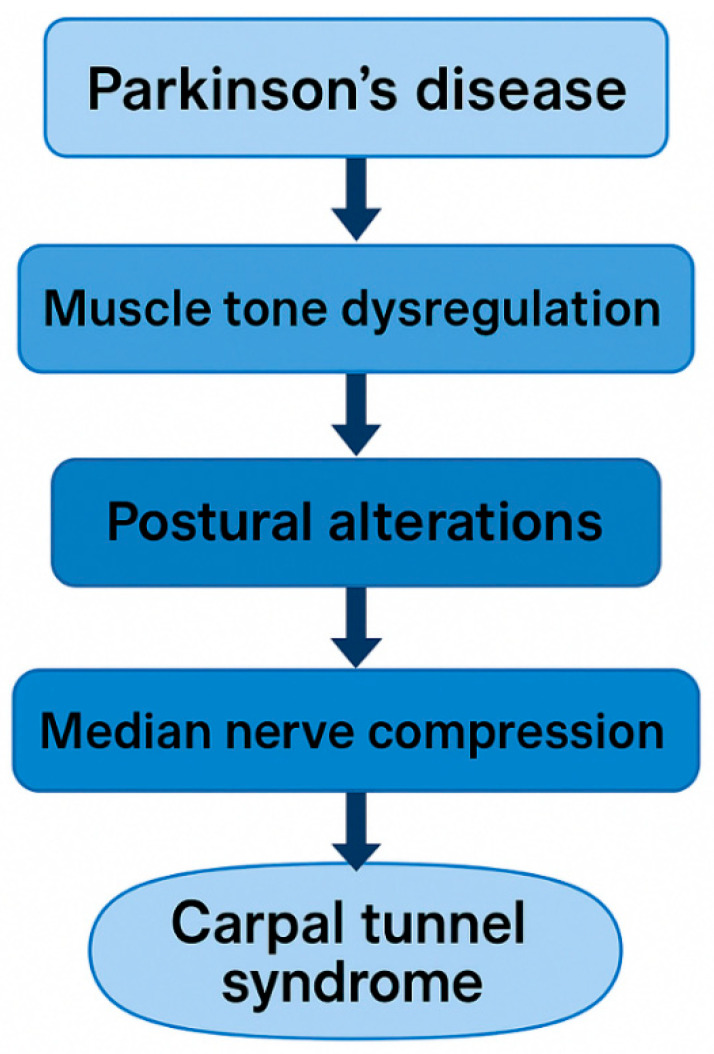
Schematic figure illustrating possible mechanistic links between PD and CTS.

Emerging evidence suggests that CTS in PD may exhibit specific clinical and electrophysiological characteristics. Yardimci et al. [32] reported that median neuropathy is relatively common in PD, often bilateral, and may correspond to the side of tremor dominance, consistent with asymmetric mechanical stress induced by tremor activity. Similarly, Yücel et al. [33] demonstrated through ultrasonography that individuals with PD have a higher CTS prevalence and an increased median nerve CSA compared to controls, emphasising the value of ultrasound for detecting subclinical involvement. Ultrasound can also visualise additional structural features relevant to CTS, including perineural fluid, tendon gliding behaviour, and dynamic nerve deformation, supporting its potential for early detection of median nerve pathology; this aligns with broader sonographic evidence highlighting the diagnostic utility of median nerve CSA and structural characteristics in CTS [48]. Other mononeuropathies, including ulnar and, more rarely, radial neuropathies, have also been reported in PD, likely reflecting cumulative postural and biomechanical stress on the upper limb. Altered central sensorimotor processing and pain modulation in PD [17] may further amplify the sensory consequences of peripheral compressive lesions, contributing to the heterogeneous and sometimes asymmetric presentation of CTS in this population. Deep-brain stimulation of the subthalamic nucleus has additionally been associated with new-onset CTS in up to 8% of patients within two years post-surgery, likely due to altered hand posture and neuromuscular control [29]. A conceptual model summarising the potential neuromechanical pathways linking Parkinson’s disease and carpal tunnel syndrome is shown in Figure 7.

Despite these findings, several limitations must be acknowledged. First, there was substantial heterogeneity in prevalence estimates (*I^2^* = 91%) due to variations in CTS diagnostic criteria and study populations. Second, the small number of included studies (*n* = 7) and overall sample size (*N* = 411) limited statistical power and increased the likelihood of small-study effects. Third, heterogeneity in CTS diagnostic methods, ranging from clinical assessment to electromyography and ultrasonography, likely contributed to inconsistent prevalence rates. Diagnostic variability, combined with inconsistent reporting of key confounders such as diabetes, BMI, and occupational factors, may further limit interpretation of the observed associations. Restricting the search to English-language studies may have introduced language bias, as potentially relevant studies published in other languages could not be evaluated at full text, reducing the completeness and representativeness of the evidence base. CTS laterality (unilateral versus bilateral involvement) was inconsistently reported, preventing stratified analyses despite its relevance to asymmetric PD motor features. A formal assessment of the certainty of the evidence (e.g., using GRADE) was not conducted due to the limited number of studies and substantial heterogeneity; therefore, the strength of the conclusions should be interpreted with caution. Finally, because the literature search concluded in April 2024, newer studies were not captured. An updated search closer to publication may identify additional eligible data and improve the precision of pooled estimates.

Future studies should adopt standardised diagnostic criteria for CTS and involve larger, multicentre cohorts to enhance generalisability. Prospective longitudinal designs are particularly needed to investigate the temporal and potentially causal relationship between PD and CTS. Subgroup analyses should examine differences by Parkinson’s disease phenotype (e.g., tremor-dominant vs. akinetic-rigid) and treatment modality, including deep brain stimulation. Future research should also systematically report CTS laterality (unilateral vs. bilateral) to determine whether patterns of involvement correspond to tremor dominance or asymmetric motor impairment in PD. Controlling for key confounders, such as diabetes, BMI, and occupational exposure, and incorporating objective outcome measures will further strengthen the quality of the evidence. Importantly, physiotherapy-focused research should investigate whether CTS in individuals with PD contributes to upper-limb disability, impaired dexterity, or reduced responsiveness to rehabilitation, thereby helping inform more targeted assessments and interventions in clinical practice.

## 5. Conclusions

In summary, this systematic review and meta-analysis provide preliminary evidence supporting a potential link between PD and CTS. Rather than a coincidental co-occurrence, CTS in PD may represent one sign of a broader neuromechanical syndrome in which central dopaminergic dysfunction interacts with peripheral biomechanical factors, such as rigidity, bradykinesia, and abnormal muscle recruitment, to increase mechanical stress along the median nerve pathway. While statistical heterogeneity and small sample sizes limit definitive conclusions, the trend toward increased CTS prevalence in PD has important clinical implications. From a functional morphology and kinesiology perspective, CTS may further degrade upper-limb movement efficiency and hand use in PD. Clinicians should therefore proactively screen for CTS symptoms, including hand paresthesia, weakness, and sensory loss, while physiotherapists should include assessment of hand function, grip strength, fine motor control, and postural alignment in routine evaluations. Future well-powered longitudinal studies are needed to clarify causality, explore interactions between central and peripheral mechanisms, and identify subgroups at greater risk (e.g., those with tremor-dominant PD or post-deep brain stimulation patients). This research will be essential for developing interdisciplinary screening protocols, evidence-based management guidelines and treatment strategies for this complex neuromechanical interaction.

## 6. Implications for Physiotherapy Practice

Clinicians managing individuals with PD should maintain a high index of suspicion for CTS, particularly in patients presenting with upper limb sensory symptoms, weakness, or fine motor difficulties. Differentiating CTS from PD-related motor dysfunction can be challenging; therefore, ultrasonography may serve as a practical, noninvasive adjunct to clinical assessment. Early detection and treatment of CTS may preserve hand function, facilitate rehabilitation engagement, and improve overall quality of life. Integrating CTS screening into standard physiotherapy assessments could enhance early referral and comprehensive care planning. Preventive management should adopt a comprehensive biomechanical approach. Early identification and correction of postural deviations, e.g., forward head posture, shoulder protraction, and asymmetries in forearm rotation, may reduce chronic mechanical stress along the median nerve pathway. Fascial and myofascial release techniques targeting the pectoralis minor, scalene muscles, and forearm flexor compartments may help restore soft-tissue mobility and alleviate tension on the brachial and median nerves. Proprioceptive and sensorimotor retraining, as outlined in PD rehabilitation programs, may improve postural control and reduce abnormal muscle co-contraction. Monitoring hand loading during functional tasks, such as gripping or repetitive manipulation, can guide ergonomic adjustments. Accordingly, screening for CTS in PD should extend beyond sensory symptoms to systematically assess posture, muscle tone, and functional hand use, thereby supporting early detection and intervention.

## Figures and Tables

**Table 1:** Baseline characteristics of included studies.

Study	Country	Design	Sample Size (N)		Age (Mean ± SD)		Male Gender N (%)		CTS Diagnostic Criteria	CTS Prevalence in PD	PD Subtype
			PD	Control	PD	Control	PD	Control			
Correa Filho et al. [34]	USA	CS	22	NA	64.5 ± 8.3	NA	10 (45.5)	NA	American Academy of Neurology	15.91%	NR
Loizon et al. [29]	France	CS	115	NA	64 ± 3.72	NA	NA	NA	American Association of Electrodiagnostic Medicine	5.22%	TD/AR/Mixed
Sengeze et al. [30]	Turkey	CS	71	NA	71.2 ± 8.6	NA	43 (60.6)	NA	Combination of clinical signs and electromyography	38.73%	TD/AR
Yang et al. [31]	South Korea	CS	42	16	64.95 ± 11.13	60.25 ± 14.67	14 (34.1)	5 (31.3)	American Association of Electrodiagnostic Medicine	7.32%	NR
Yardimci et al. [32]	Turkey	CS	31	32	66.48 ± 10.67	62.03 ± 10.4	31 (41.9)	10 (31.3)	Combination of clinical signs and electromyography	16.13%	NR
Han et al. [12]	South Korea	CS	33	NA	67.9 ± 7.49	NA	16 (49)	NA	Combination of clinical signs and electromyography	10.61%	TD
Yucel et al. [33]	Turkey	CS	29	20	66.5 ± 9.7	60.3 ± 9.1	17 (59)	9 (45)	Combination of clinical signs and electromyography	24.52%	TD

Abbreviations: CS: Cross-sectional design, N: Number, CTS: Carpal tunnel syndrome, SD: Standard deviation, PD: Parkinson’s disease, NR = Not Reported, TD = Tremor-Dominant, AR = Akinetic-Rigid, Mixed = Mixed Phenotype (features of both TD and AR).

**Table 2:** Pooled prevalence of CTS in examined PD wrists.

Study	Country	CTS in PD Patients			CTS in Control		
		Event	Total	Percentage	Event	Total	Percentage
Correa Filho et al. [34]	USA	7	44	15.91	NA	NA	NA
Loizon et al. [29]	France	12	230	5.22	NA	NA	NA
Şengeze et al. [30]	Turkey	55	142	38.73	NA	NA	NA
Yang et al. [31]	South Korea	6	82	7.32	0	31	0
Yardimci et al. [32]	Turkey	10	62	16.13	0	62	0
Han et al. [12]	South Korea	7	66	10.61	NA	NA	NA
Yucel et al. [33]	Turkey	13	53	24.52	7	36	19.44

Abbreviations: CTS: Carpal tunnel syndrome, PD: Parkinson’s disease, NA: not available.

## Data Availability

The data generated and analysed for the meta-analyses are available from the corresponding author upon reasonable request.

## Supplementary Materials

The following supporting information can be downloaded at: https://www.mdpi.com/article/10.3390/jfmk11010066/s1, Supplementary File S1: Categorisation of search terms used to identify studies on carpal tunnel syndrome and associated risk in individuals with Parkinson’s disease.

## Abbreviations

The following abbreviations are used in this manuscript:

AAEM	American Association of Electrodiagnostic Medicine
CI	Confidence interval
CSA	Cross-sectional area
CTS	Carpal tunnel syndrome
DBS	Deep brain stimulation
EMG	Electromyography
GRADE	Grading of Recommendations Assessment, Development and Evaluation
MRI	Magnetic resonance imaging
NA	Not available
NIH	National Institutes of Health
PD	Parkinson’s disease
PRISMA	Preferred Reporting Items for Systematic Reviews and Meta-Analyses
RD	Risk difference
RR	Risk ratio
