# Supplementary material for: Prevalence and Risk of Carpal Tunnel Syndrome in Parkinson’s Disease: A Systematic Review and Meta-Analysis
Source: J Funct Morphol Kinesiol. 2026 Feb 2;11(1):66. doi: 10.3390/jfmk11010066 (PMC12921936; doi:10.3390/jfmk11010066)
Supplement: Supplementary file 1 [file jfmk-11-00066-s001.zip › jfmk-4126814-supplementary.pdf]

Supplementary File S1. Categorisation of search terms used to identify studies on Carpal Tunnel Syndrome and associated risk in individuals with Parkinson's Disease.

Database	Search strategy	Number of results
PubMed	((Parkinson Disease[Title/Abstract] OR Parkinson's Disease[Title/Abstract] OR Lewy Body Parkinson Disease[Title/Abstract] OR Idiopathic Parkinson Disease[Title/Abstract] OR Parkinsonism[Title/Abstract] OR PD[Title/Abstract]) AND (Carpal Tunnel Syndrome[Title/Abstract] OR Compression Neuropathy[Title/Abstract] OR Median Neuropathy[Title/Abstract] OR Median Nerve Neuralgia[Title/Abstract] OR Entrapment Neuropathy[Title/Abstract] OR Thenar Amyotrophy Carpal Origin[Title/Abstract]))<br>**Fields searched:** Title and Abstract	1261
Scopus	TITLE-ABS-KEY (("carpal tunnel syndrome") OR ("compression neuropathy") OR ("median neuropathy") OR ("median nerve neuralgia") OR ("entrapment neuropathy") OR ("thenar amyotrophy carpal origin")) AND TITLE-ABS-KEY (("Parkinson disease") OR ("Parkinson's disease") OR ("Lewy body Parkinson disease") OR ("Idiopathic Parkinson disease") OR ("Parkinsonism") OR ("PD"))<br>**Fields searched:** *Title, Abstract, Keywords*	690
Embase	('carpal tunnel syndrome'/exp OR 'median neuropathy at the wrist'/exp OR 'CTS') AND ('parkinson disease'/exp OR 'parkinson's disease' OR 'parkinsonism') AND ('prevalence'/exp OR 'epidemiology'/exp OR 'incidence'/exp OR 'cross-sectional study'/exp OR 'case-control study'/exp OR 'cohort study'/exp)<br>**Fields searched:** Emtree terms (exploded), Title, Abstract	49
Web of Science (WOS)	TS=("Carpal Tunnel Syndrome" OR "Median Neuropathy at the Wrist" OR "CTS") AND TS=("Parkinson Disease" OR "Parkinson's Disease" OR "Parkinsonism") AND TS=("Prevalence" OR "Epidemiology" OR "Incidence" OR "Cross-Sectional Studies" OR "Case-Control Studies" OR "Cohort Studies")<br>**Fields searched:** Topic (TS = Title, Abstract, Author Keywords, and Keywords Plus)	59
Cochrane	((Carpal tunnel syndrome) OR (Median Neuropathy) OR	217

Central Register of Controlled Trials (CENTRAL)	(Compression Neuropathy) OR (Entrapment Neuropathy) OR (CTS)) AND ((Parkinson Disease) OR (Parkinson's Disease) OR (Parkinsonism) OR (Paralysis Agitans) OR (PD))<br>**Fields searched:** Title, Abstract, Keyword (word variations automatically searched)	
